# Distinctive Pattern of Cognitive Disorders During Multiple Sclerosis Relapse and Recovery Based on Computerized CANTAB Tests

**DOI:** 10.3389/fneur.2019.00572

**Published:** 2019-06-04

**Authors:** Natasa Giedraitiene, Gintaras Kaubrys

**Affiliations:** Clinic of Neurology and Neurosurgery, Institute of Clinical Medicine, Faculty of Medicine, Vilnius University, Vilnius, Lithuania

**Keywords:** multiple sclerosis, relapse, cognition, neuropsychological test, CANTAB

## Abstract

**Background:** Cognition may be affected at least as seriously as physical function during multiple sclerosis (MS) relapse, however MS relapse related cognitive disorders are still underdiagnosed and poorly characterized. The limited number of paper-pencil tests were used for assessment, and nevertheless, some significant changes were found. Unlike the paper-pencil tests, computerized batteries and tests are more sensitive and highly standardized, produce instant scoring and can minimize the learning and practice effects on follow-up. We investigated the cognition during MS relapse with the Cambridge Neuropsychological Test Automated Battery (CANTAB), which has shown sensitivity to cognitive dysfunction across different clinical groups, including patients with MS.

**Objective:** The objective of the study was to assess the cognitive functions with CANTAB battery in MS patients during relapse, in stable MS patients, and healthy controls, and to establish the timing and pattern of cognitive recovery after relapse.

**Methods:** Sixty relapsing, thirty stable MS patients, and thirty controls were assessed with CANTAB. The relapse group was assessed during multiple sclerosis relapse and 1 and 3 months after the first assessment.

**Results:** The score of the difficult task of spatial planning was worse in MS relapse group than in MS stable group (*p* < 0.05). The scores of medium difficulty tasks of spatial planning, episodic visual recall and working memory were worse in the relapse group than in the control group (*p* < 0.05), while in stable MS and control groups, the scores of these tasks didn't differ. The most significant improvement of speed of response, spatial planning, episodic visual recall memory and spatial working memory, was established at 1 month after the first assessment, additional improvement of spatial planning and working memory was observed at 3 months after the first assessment.

**Conclusions:** The results of this study indicate that cognitive function is affected during MS relapse. The difficult task of CANTAB battery, which assesses the spatial planning, showed MS relapse related cognitive dysfunction. The changes in scores of episodic visual recall and working memory may be related to MS relapse. A significant improvement in the speed of response, spatial planning, episodic visual recall and working memory was established at 1 month after MS relapse. The additional improvement in spatial planning for the most difficult task and working memory was observed at 3 months after MS relapse. It may be possible that the practice effect had the impact on the improvement of cognitive scores that was noted in relapsing MS patients.

## Introduction

The impairment of cognitive functions is found in up to 70% of patients with multiple sclerosis (MS) (1). It has been demonstrated at all stages and in all subtypes of the disease. However, the more severe levels of cognitive impairment (CI) tend to occur in the progressive phase of the disease, but typically it is only loosely related to disease duration and physical disability (1, 2). Almost all types of cognitive deficits can be found in MS, but the typical profile consists of disorders in information processing speed, working memory, and executive function impairments, while language and general intelligence are commonly preserved (1, 2). Cognitive impairment is often the leading predictor of occupational disability in MS patients even when the physical disability is quite low (3).

Recently, a great deal of attention was given to cognition during MS relapse. It has been established that cognition could also be affected during relapse and so-called “cognitive-relapses” (with or without physical disability symptoms) are hypothesized to occur during MS relapse. It seems that CI during relapse is even more common than previously was thought (4, 5). CI is an important determinant of disability in MS, and that is the reason why cognitive assessment was suggested to be included in the treatment efficacy evaluations in recent years. However, due to the limitations of traditional paper-pencil cognitive tests, cognitive relapses are still underdiagnosed or incompletely evaluated in MS patients (6).

Computerized batteries and tests are sensitive, highly standardized, reliable, easy to use, produce instant scoring and can minimize the learning and practice effects on follow-up (7–10). Recently, cognitive impairment in MS patients was detected using Cambridge Neuropsychological Test Automated Battery (CANTAB) (11, 12). This computerized cognitive testing battery is widely used, very well validated and has reliable neuropsychological battery (13, 14). It consists of a number of computerized tests that can be administered *via* touchscreen platforms to measure distinct cognitive domains (15). CANTAB tasks have shown sensitivity to cognitive dysfunction and have been widely used across different clinical groups (16–19), including MS patients (11). CANTAB results from MS studies, which have confirmed that CI occurs across a range of domains among MS patients (11, 12), while the most frequently impaired domain was executive function. The impairment of executive function was present in more than half of MS patients (11).

From the clinical and scientific point of view, it is useful to assess the neurocognitive status along with other aspects of neurological disability during MS relapse and to improve the understanding of the clinically meaningful changes in cognition outcomes that may occur as a result of neurological worsening or response to treatment. While a number of cognitive batteries have been developed specifically for use in patients with MS, only some of the paper-pencil tests were used for assessment during MS relapse and significant changes were found (4, 5). However, there are no data about the assessment of cognition with CANTAB during MS relapse and after relapse.

The objective of our study was to assess the cognitive functions with CANTAB battery in MS patients during relapse, in stable MS patients and healthy controls, and to establish the timing and pattern of cognitive recovery after relapse.

## Materials and Methods

### Subjects

One hundred and twenty subjects were enrolled in the prospective study at the Multiple Sclerosis Center Vilnius University Hospital Santaros Klinikos. We recruited 60 relapsing (MSr) and 30 stable (MSs) patients with clinically definite MS according to the McDonald 2010 criteria and 30 healthy controls (control group, CG) matched according to age, education, and gender.

Relapse group was assessed during relapse (MSr1), then 1 month (MSr2) and 3 months (MSr3) after the first assessment. All patients in relapse and stable groups had relapsing-remitting MS.

Inclusion criteria for both MS groups comprised: no relapse or steroid treatment within in the past 3 months and the first symptoms of relapse in the relapse group were separated from the previous relapse by at least 3 months. Exclusion criteria for MS patients were neurologic or psychiatric disorders (such as seizure disorders, major depressive disorder, cerebral palsy, bipolar disorder, and others) that could mimic MS, moderate or severe fatigue, anxiety or depression and any medication consumption that could compromise the cognitive functions. No patient had optic neuritis or dominant extremity weakness or significant ataxia. The CG included healthy persons with no history of any cognitive dysfunction, fatigue or depression, or any medication consumptions and drug or alcohol abuse in the past. All participants had sufficient sight and hearing for compliance with the study assessment.

### Neurological and Comorbidity Assessment

Neurological assessment was performed in all MS patients. Neurological disability was assessed with the Expanded Disability Status Scale (EDSS). MS relapses were diagnosed in the cases of new neurological symptoms or worsening of previous symptoms appearance that lasted for at least 24 h and occurred in the absence of fever or any other known trigger (20, 21). Fatigue was evaluated with the Fatigue Severity Scale (22), depression with the Hospital Anxiety and Depression Scale (23). Patients were excluded if diagnosed with severe fatigue, anxiety or depression. Cut-off points of 4/9 and 12/21 were identified as indicative of possible fatigue and depression, respectively.

### Neuropsychological Assessment

All subjects were assessed with the CANTAB in the same room and by the same person. CANTABeclipse™ (Cambridge Cognition Ltd., United Kingdom) software on touch screen tablet computer with press pad was used for testing. Relapse group was examined during relapse, 1 and 3 months after the first assessment. The visits were performed within the allowed visit window of ±3 days. Assessment during relapse (MSr1 group) was performed before the relapse treatment. The relapses were treated by the treating physician according to the standard clinical practice. The cognition-evaluating physician had no influence on the relapse treatment options. Assessment at 1 month (MSr2 group) was performed at 1 month (±3 days) after the first assessment, and assessment at 3 months (MSr3 group) was performed at 3 months after the first assessment. To minimize the practice effect the alternative versions of CANTAB were used.

Four CANTAB tests were included in the assessment (13). The tests measured four different cognitive domains and those domains that are most commonly affected in MS. All participants were assessed with:

1. **Reaction time (RTI) test**—measures the subject's speed of response to a visual target (13).
2. **One touch stockings of Cambridge (OTS) test**—test gives the measure of frontal lobe function (13).
3. **Paired Associates Learning (PAL) test**—assesses visual memory, new learning and is primarily sensitive to changes in medial temporal lobe functioning (13).
4. **Spatial Working Memory (SWM) test**—is a test of the subject's ability to retain spatial information and to manipulate remembered items in working memory. This test is a sensitive measure of frontal lobe and executive dysfunction (13).

### Statistical Analysis

Data were analyzed using SPSS statistical software (version 20.0 for Windows). Descriptive statistics are presented as the mean (m) and standard deviation (±SD). Student's *t*-test was used to compare means of the same variables in two groups when their distribution was normal. Categorical variables were analyzed using the chi-square or Fisher's exact test. To check the normality of distribution of quantitative variables, the Shapiro-Wilk test was used. One-way analysis of variance (ANOVA) was used to compare the means of relapse, stable groups and CG. To determine the significance of pairwise comparisons in one-way ANOVA, Bonferroni's *post hoc* test (for equal variances, Levene's test *p* > 0.05) or Tamhane's test (for unequal group variances, Levene's test *p* < 0.05) was used. Repeated measures ANOVA (rmANOVA) was used when assessing the data of the relapse group at different time points (relapse at 1 and 3 months after the first assessment). Greenhouse-Geisser correction was used to assure the equality of the variances of the differences between all possible pairs of within-subject conditions. Effect size was evaluated using the Cohen's d: Cohen's *d* = 0.2, 0.5, and 0.8 corresponded to small, medium and large effects. All analyses were two-tailed, *p* < 0.05 was considered statistically significant.

## Results

### Sample Characteristics

Relapse and stable groups and healthy controls (CG) were matched on the demographic characteristics (Table 1).

**Table 1:** Demographic and clinical characteristics in MS patients and control group.

	**MSr group**	**MSs group**	**CG**	**Test**
Number of subjects, *N*	60	30	30	–
Gender Men/Women, N	21/39	13/17	12/18	χ2 = 0.64 *p* = 0.73
Age (years) Mean ± SD (Range)	38.43 ± 9.59 (18–61)	37.47 ± 10.34 (22–59)	37.63 ± 9.51 (24–56)	ANOV *F* = 0.13 *p* = 0.88
Education (years) Mean ± SD	14.80 ± 2.26	14.88 ± 2.80	15.47 ± 1.79	ANOVA *F* = 0.88 *p* = 0.42
EDSS 3 mth before relapse	3.59 ± 1.29	3.25 ± 1.19	-	*p* = 0.24
EDSS during relapse^*^	5.03 ± 1.00	3.28 ± 1.18	-	p < 0.001
EDSS 1st mth after the first asessment^*^	3.81 ± 1.27	3.32 ± 1.19	-	*p* = 0.80
EDSS 3rd mth after the first assessment^*^	3.76 ± 1.26	3.30 ± 1.21	-	*p* = 0.90

MSr, relapse group; MSs, stable group; CG, control group; EDSS, Expanded Disability Status Scale.

* *Date of relapse in MSr group*.

The mean duration of the disease in relapse group was 8.94 ± 7.21 and in stable group was 8.30 ± 7.50 (*p* = 0.70). The period of remission in the relapse group, before relapse under assessment, was 23.22 ± 23.80 [95% confidence interval 17.07–29.37 months] and in stable group was 30.23 ± 29.18 [95% confidence interval 19.31–41.10 months] (*p* = 0.23).

MS relapse in 29 (48.3%) relapsing patients was treated with intravenous methylprednisolone, in 6 (10.0%) patients—with plasma exchange and in 25 (41.7 %)—with methylprednisolone and plasma exchange. All MS patients received disease modifying therapy. Overall, five medications were used and there was no significant difference in the distribution of medications between MSr and MSr groups (*p* > 0.05).

### Assessment With CANTAB Tests

The cognitive performance on CANTAB was compared between relapse group, stable group and CG. CANTAB testing lasted 46.26 ± 13.19 min. (from 22.77 up to 102.63 min.) in all participants. In MS patients (relapsing and stable) CANTAB testing lasted significantly longer than in CG (accordingly 51.02 ± 14.24, 45.08 ± 11.20, and 37.90 ± 7.46), however, there were no significant differences between testing time in MSr and MSs groups (ANOVA *F* = 11.89, *p* < 0.001).

### The Scores of CANTAB Tests in MS Relapse Group, Stable MS Group, and Healthy Controls

The mean scores of simple reaction time (RTI-SR), five-choice movement time (RTI-FM) and five-choice reaction time (RTI-FR) were significantly higher in MS patients than in CG (*p* < 0.05), while in relapse and stable groups there were no significant differences between mean scores of above mentioned values (*p* > 0.05) (Table 2).

**Table 2:** RTI test scores in MS relapse group, stable MS group, and healthy controls.

**Test**	**MSr group** **(N-60)**	**MSs group** **(N-30)**	**CG** **(N-30)**	**ANOVA**	***Post hoc***
RTI-FM	569.30 ± 182.95	563.00 ± 316.74	359.75 ± 76.25	*F* = 11.22; p < 0.001	CG < MSs,MSr^#^, MSs = MSr^**^^##^
RTI-FR	416.73 ± 99.74	377.73 ± 68.67	332.10 ± 47.85	*F* = 10.80; p < 0.001	CG < MSs,MSr MSs = MSr^**^
RTI-SR	392.95 ± 95.46	368.29 ± 88.72	305.98 ± 43.06	*F* = 10.82; p < 0.001	CG < MSs,MSr MSs = MSr^**^

MSr, relapse group; MSs, stable group; CG, control group; RTI, reaction time; RTI-FM, five-choice movement time of RTI; RTI-FR, five-choice reaction time of RTI; RTI-SR, simple reaction time of RTI.

Low score denotes better cognition.

** Tamhane test was used for post hoc analysis.

# CG < MSr indicates that the means of groups CG and MSr significantly differ.

## *MSs = MSr indicates that the means of groups MSs and MSr do not differ significantly*.

The mean choice to correct of 1, 5, and 6 moves (OTS-MC 1/5/6) of OTS test were significantly lower in CG than in relapse group (*p* < 0.05), while OTS-MC of 2, 3, and 4 moves did not differ in CG, MSr, and MSs groups (*p* > 0.05) (Table 3).

**Table 3:** OTS test scores in MS relapse group, stable MS group, and healthy controls.

**Test**	**MSr group** **(N-60)**	**MSs group** **(N-30)**	**CG** **(N-30)**	**ANOVA**	***Post hoc***
OTS-MC1	1.09 ± 0.16	1.03 ± 0.09	1.03 ± 0.08	*F* = 3.68; *p* = 0.028	CG < MSr^**^^#^ CG = MSs^##^ MSs = MSr
OTS-MC2	1.12 ± 0.16	1.11 ± 0.17	1.04 ± 0.09	*F* = 3.00; *p* = 0.054	CG = MSs = MSr^*^
OTS-MC3	1.14 ± 0.21	1.11 ± 0.14	1.06 ± 0.11	*F* = 2.27; *p* = 0.11	CG = MSs = MSr^*^
OTS-MC4	1.39 ± 0.43	1.33 ± 0.35	1.22 ± 0.32	*F* = 2.06; *p* = 0.13	CG = MSs = MSr^*^
OTS-MC5	1.55 ± 0.41	1.48 ± 0.42	1.33 ± 0.27	*F* = 3.37; *p* = 0.038	CG < MSr^*^ CG = MSs MSs = MSr
OTS-MC6	2.09 ± 0.78	1.73 ± 0.50	1.45 ± 0.36	*F* = 10.67; p < 0.001	CG,MSs < MSr, CG = MSs^**^

MSr, relapse group; MSs, stable group; CG, control group; OTS, One touch stockings of Cambridge; OTS-MC1/2/3/4/5/6, mean choice to correct of 1/2/3/4/5/6 moves of OTS.

Low score denotes better cognition.

* Bonferroni test was used for post hoc analysis.

** Tamhane test was used for post hoc analysis.

# CG < MSr indicates that the means of groups CG and MSr significantly differ.

## *CG = MSs indicates that the means of groups CG and MSs do not differ significantly*.

Total errors of PAL test (PAL-TE) were significantly lower in CG than in MSs and MSr groups (*p* < 0.001). The results of PAL-TE at stages with different pattern numbers were significantly worse and the differences were greater between all three groups the more difficult was the task: at the 1, 2, and 3 figure stages there were no significant differences between all three groups (*p* > 0.05), at the 6-figure stage MSr group made significantly more errors than CG and MSs group (*p* < 0.05), and at the 8-figure stage both MSr and MSs groups made significantly more errors than CG (*p* < 0.05), while the results in MSs and MSr groups didn't differ (Table 4).

**Table 4:** PAL test scores in MS relapse group, stable MS group, and healthy controls.

**Test**	**MSr group** **(N-60)**	**MSs group** **(N-30)**	**CG** **(N-30)**	**ANOVA**	***Post hoc***
PAL-TE	17.63 ± 13.08	12.70 ± 9.60	6.77 ± 6.06	*F* = 10.15; p < 0.001	CG < MSs,MSr^#^, MSs = MSr^**^^##^
PAL-TE1	0	0	0	-	CG = MSs = MSr^*^
PAL-TE2	0.18 ± 0.62	0.23 ± 0.57	0.13 ± 0.51	*F* = 0.22; *p* = 0.80	CG = MSs = MSr^*^
PAL-TE3	1.17 ± 1.91	0.90 ± 1.40	0.63 ± 1.35	*F* = 1.06; *p* = 0.35	CG = MSs = MSr^*^
PAL-TE6	5.52 ± 5.04	3.27 ± 4.51	1.77 ± 1.98	*F* = 8.08; *p* = 0.001	CG < MSr^**^ CG = MSs MSs = MSr
PAL-TE8	10.82 ± 8.99	8.47 ± 6.86	4.23 ± 3.64	*F* = 7.79; *p* = 0.001	CG < MSs,MSr MSs = MSr^**^

MSr, relapse group; MSs, stable group; CG, control group; PAL, Paired Associates Learning; PAL-TE, total errors of PAL; PAL-TE1/2/3/6/8, total errors at the 1-, 2-, 3-, 6- and 8-figure stages of PAL.

Low score denotes better cognition.

* Bonferroni test was used for post hoc analysis.

** Tamhane test was used for post hoc analysis.

# CG < MSr indicates that the means of groups CG and MSr significantly differ.

## *MSs = MSr indicates that the means of groups MSs and MSr do not differ significantly*.

Total errors of SWM test (SWM-TE) were significantly lower in CG than in both MS groups (*p* < 0.001), while the results of these values in MSr and MSs groups did not differ (*p* > 0.05). Total errors for four boxes (SWM-TE4) did not differ in all three groups (*p* > 0.05). As in the cases of OTS and PAL, the differences of SWM-TE in stages with a different number of boxes in all three groups were more obvious the more difficult was the task: the result of SWM-TE4 didn't differ between all three groups (*p* > 0.05), SWM-TE6 were significantly lower in CG than in MSr group (*p* < 0.001), while the results of MSs group did not differ neither from CG, nor from MSr group (*p* > 0.05); SWM-TE8 were significantly lower in CG than in MSs and MSr groups (*p* < 0.001), while in MSs and MSr groups the results did not differ (*p* > 0.05) (Table 5).

**Table 5:** SWM test scores in MS relapse group, stable MS group, and healthy controls.

**Test**	**MSr group (N-60)**	**MSs group (N-30)**	**CG (N-30)**	**ANOVA**	***Post hoc***
SWM-TE	33.03 ± 19.94	23.40 ± 19.02	11.37 ± 11.08	*F* = 14.85; *p* < 0.001	CG < MSs,MSr^#^ MSs = MSr^**^^##^
SWM-TE4	1.12 ± 1.94	0.90 ± 1.77	0.23 ± 0.57	*F* = 2.86; *p* = 0.061	CG = MSs = MSr^*^
SWM-TE6	8.58 ± 8.60	4.90 ± 6.31	1.93 ± 2.66	*F* = 9.53; *p* < 0.001	CG < MSr^**^ CG = MSs MSs = MSr
SWM-TE8	23.18 ± 12.53	17.40 ± 12.35	9.13 ± 9.70	*F* = 14.17; *p* < 0.001	CG < MSs,MSr MSs = MSr^*^

MSr, relapse group; MSs, stable group; CG, control group; SWM, Spatial Working Memory; SWM-TE, total errors of SWM; SWM-TE4/6/8, total errors for 4, 6 or 8 boxes of SWM.

Low score denotes better cognition.

* Bonferroni test was used for post hoc analysis.

** Tamhane test was used for post hoc analysis.

# CG < MSr indicates that the means of groups CG and MSr significantly differ.

## *MSs = MSr indicates that the means of groups MSs and MSr do not differ significantly*.

### The Scores of CANTAB Tests During MS Relapse and Recovery Stage

MS relapse group was assessed three times (during relapse, at 1 month and at 3 months after the first assessment. The means of most RTI test scores were significantly lower at 1 month after the first assessment compared to during relapse: the mean score of RTI-SR–by 33.37 ms, RTI-FM–by 68.33 ms, and RTI-FR–by 30.69 ms. shorter at 1 month after the first assessment than during relapse (for all scores *p* < 0.001), however, the mean scores at 1 and 3 months after the first assessment did not differ (*p* > 0.05) (Table 6).

**Table 6:** RTI test scores during and after MS relapse.

**Test**	**MSr1 group** **(N-60)**	**MSr2 group** **(N-60)**	**MSr3 group** **(N-60)**	**ANOVA**	***Post hoc***	**Cohen's *d***
RTI-FM	569.30 ± 182.95	500.97 ± 135.12	480.69 ± 170.50	*F* = 12.42; *p* < 0.001^*^	MSr1>MSr2,MSr3 MSr2 = MSr3	MSr1_MSr2 = 0.43 MSr2_MSr3 = 0.17
RTI-FR	416.73 ± 99.74	386.04 ± 74.08	374.66 ± 64.95	*F* = 19.85; *p* < 0.001^*^	MSr1>MSr2,MSp3 MSr2 = MSr3	MSr1_MSr2 = 0.35 MSr2_MSr3 = 0.16
RTI-SR	392.95 ± 95.46	359.58 ± 79.62	352.46 ± 78.79	*F* = 11.42; *p* < 0.001	MSr1>MSr2,MSr3 MSr2 = MSr3	MSr1_MSr2 = 0.38 MSr2_MSr3 = 0.09

MSr1, relapse group during relapse; MSr2, relapse group at 1 month after the first assessment; MSr3, relapse group at 3 months after the first assessment; RTI, reaction time; RTI-FM, five-choice movement time of RTI; RTI-FR, five-choice reaction time of RTI; RTI-SR, simple reaction time of RTI.

Low score denotes better cognition.

* - *Repeated Measures Analysis of Variance (rmANOVA), Greenhouse-Geisser correction for violation of sphericity was used*.

The differences between results of OTS-MC in tasks with a different number of moves were more obvious the more difficult was the task: the result of OTS-MC of 3 moves was significantly lower at 3 months after the first assessment than during relapse, OTS-MC of 4 and 5 moves were significantly lower at 1 month after the first assessment, while OTS-MC of 6 moves was significantly lower at 1 and at 3 months after the first assessment than during relapse (*p* < 0.05) (Table 7).

**Table 7:** OTS test scores during and after MS relapse.

**Test**	**MSr1 group** **(N-60)**	**MSr2 group** **(N-60)**	**MSr3 group** **(N-60)**	**ANOVA**	***Post hoc***	**Cohen's *d***
OTS-MC1	1.09 ± 0.16	1.03 ± 0.86	1.03 ± 0.09	*F* = 5.85; *p* = 0.004^*^	MSr1>MSr2,MSr3 MSr2 = MSr3	MSr1_MSr2 = 0.46 MSr2_MSr3 = 0
OTS-MC2	1.12 ± 0.16	1.13 ± 0.18	1.10 ± 0.15	*F* = 0.41; *p* = 0.64^*^	MSr1 = MSr2 = MSr3	MSr1_MSr2 = 0.06 MSr2_MSr3 = 0.18
OTS-MC3	1.14 ± 0.21	1.08 ± 0.14	1.07 ± 0.12	*F* = 4.35; *p* = 0.027^*^	MSr1>MSr3 MSr1 = MSr2 MSr2 = MSr3	MSr1_MSr2 = 0.34 MSr2_MSr3 = 0.08
OTS-MC4	1.39 ± 0.43	1.18 ± 0.24	1.18 ± 0.31	*F* = 13.50; *p* < 0.001^*^	MSr1>MSr2,MSr3 MSr2 = MSr3	MSr1_MSr2 = 0.60 MSr2_MSr3 = 0
OTS-MC5	1.55 ± 0.41	1.36 ± 0.32	1.40 ± 0.32	*F* = 6.81; *p* = 0.004^*^	MSr1>MSr2,MSr3 MSr2 = MSr3	MSr1_MSr2 = 0.52 MSr2_MSr3 = 0.13
OTS-MC6	2.09 ± 0.78	1.75 ± 0.58	1.60 ± 0.45	*F* = 21.04; *p* < 0.001^*^	MSr1>MSr2>MSr3	MSr1_MSr2 = 0.49 MSr2_MSr3 = 0.29

MSr1, relapse group during relapse; MSr2, relapse group at 1 month after the first assessment; MSr3, relapse group at 3 months after the first assessment; OTS, One touch stockings of Cambridge; OTS-MC 1/2/3/4/5/6, mean choice to correct of 1/2/3/4/5/6 moves of OTS.

Low score denotes better cognition.

* - *Repeated Measures Analysis of Variance (rmANOVA), Greenhouse-Geisser correction for violation of sphericity was used*.

PAL-TE were significantly higher during relapse than at 1 month after the assessment (*p* < 0.05) and the results of these values did not differ at 3 months and 1 month after the first assessment in the relapse group (*p* > 0.05) (Table 8). The differences between results of PAL-TE in tasks with different figure stages were more obvious the more difficult was the task: the result of PAL-TE1 and PAL-TE2 didn’t differ during relapse (*p* > 0.05), at 1 and 3 month after the first assessment, the result of PAL-TE3 was significantly lower at 3 months after the first assessment than during relapse, the results of PAL-TE6 and PAL-TE8 were significantly lower at 1 and 3 month after the first assessment than during relapse in relapse group (*p* < 0.05).

**Table 8:** PAL test scores during and after MS relapse.

**Test**	**MSr1 group** **(N-60)**	**MSr2 group** **(N-60)**	**MSr3 group** **(N-60)**	**ANOVA**	***Post hoc***	**Cohen's *d***
PAL-TE	17.63 ± 13.08	10.05 ± 8.28	9.05 ± 8.59	*F* = 35.76; *p* < 0.001^*^	MSr1>MSr2,MSr3 MSr2 = MSr3	MSr1_MSr2 = 0.69 MSr2_MSr3 = 0.12
PAL-TE1	0	0	0	-	MSr1 = MSr2 = MSr3	-
PAL-TE2	0.18 ± 0.62	0.13 ± 0.47	0.10 ± 0.40	*F* = 0.45; *p* = 0.60^*^	MSr1 = MSr2 = MSr3	MSr1_MSr2 = 0.09 MSr2_MSr3 = 0.07
PAL-TE3	1.17 ± 1.91	0.60 ± 1.21	0.62 ± 1.62	*F* = 3.50; *p* = 0.04^*^	MSr1>MSr3 MSr1 = MSr2 MSr2 = MSr3	MSr1_MSr2 = 0.36 MSr2_MSr3 = 0.01
PAL-TE6	5.52 ± 5.04	2.55 ± 2.91	2.50 ± 3.33	*F* = 17.63; *p* < 0.001^*^	MSr1>MSr2,MSr3 MSr2 = MSr3	MSr1_MSr2 = 0.72 MSr2_MSr3 = 0.01
PAL-TE8	10.82 ± 8.99	6.77 ± 6.47	5.83 ± 5.82	*F* = 15.80; *p* < 0.001^*^	MSr1>MSr2,MSr3 MSr2 = MSr3	MSr1_MSr2 = 0.52 MSr2_MSr3 = 0.14

MSr1, relapse group during relapse; MSr2, relapse group at 1 month after the first assessment; MSr3, relapse group at 3 months after the first assessment; PAL, Paired Associates Learning; PAL-TE, total errors of PAL; PAL-TE1/2/3/6/8, total errors at the 1-, 2-, 3-, 6- and 8-figure stages of PAL.

Low score denotes better cognition.

* - *Repeated Measures Analysis of Variance (rmANOVA), Greenhouse-Geisser correction for violation of sphericity was used*.

SWM-TE were significantly higher in MSr1 than in MSr2 group and were significantly higher in MSr2 than in MSr3 group (*p* < 0.001). Although the results of SWM-TE4 were relatively low (ranging from 0.48 up to 1.12 errors), however, significant differences were also found—SWM-TE4 were significantly lower in MSr3 than MSr1 group (*p* < 0.05). SWM-TE6 were significantly higher during relapse and at 1 month after the first assessment than at 3 months after the first assessment (*p* < 0.001). SWM-TE8 were significantly higher during relapse than at 1 and 3 months after the first assessment (*p* < 0.001), while the results at 1 and 3 months did not differ (*p* > 0.05) (Table 9, see Supplementary Material).

**Table 9:** SWM test scores during and after MS relapse.

**Test**	**MSr1 group** **(N-60)**	**MSr2 group** **(N-60)**	**MSr3 group** **(N-60)**	**ANOVA**	***Post hoc***	**Cohen's *d***
SWM-TE	33.03 ± 19.94	25.37 ± 21.72	21.27 ± 19.00	*F* = 19.45; *p* < 0.001^*^	MSr1>MSr2>MSr3	MSr1_MSr2 = 0.37 MSr2_MSr3 = 0.20
SWM-TE4	1.12 ± 1.94	0.97 ± 2.12	0.48 ± 1.24	*F* = 4.30; *p* = 0.018	MSr1>MSr3 MSr1 = MSr2 MSr2 = MSr3	MSr1_MSr2 = 0.07 MSr2_MSr3 = 0.29
SWM-TE6	8.58 ± 8.60	6.90 ± 8.14	4.97 ± 6.50	*F* = 10.22; *p* < 0.001^*^	MSr1,MSr2>MSr3 MSr1 = MSr2	MSr1_MSr2 = 0.20 MSr2_MSr3 = 0.26
SWM-TE8	23.18 ± 12.53	17.50 ± 15.04	15.82 ± 12.97	*F* = 11.65; *p* < 0.001^*^	MSr1>MSr2,MSr3 MSr2 = MSr3	MSr1_MSr2 = 0.41 MSr2_MSr3 = 0.12

MSr1, relapse group during relapse; MSr2, relapse group at 1 month after the first assessment; MSr3, relapse group at 3 months after the first assessment; SWM, Spatial Working Memory; SWM-TE, total errors of SWM; SWM-TE4/6/8, total errors for 4, 6 or 8 boxes of SWM.

Low score denotes better cognition.

* - *Repeated Measures Analysis of Variance (rmANOVA), Greenhouse-Geisser correction for violation of sphericity was used*.

## Discussion

Recently, it was established that cognition may be affected during MS relapse (4–6). The cognitive impairment during relapse is quite common and more frequent than was previously thought (4, 5). Most likely, the main difficulty in the assessment of cognition during relapse is the lack of suitable and sensitive assessment methods of cognition. The CANTAB has previously been shown to be sensitive to cognitive deficits in patients with MS (11, 12). We assessed the cognition with CANTAB tests in relapse and stable MS patients and healthy controls. We decided to examine both relapse and stable MS patients as assessment and differentiation of cognitive impairment in these patients separately could help to detect the clinically meaningful changes that can occur during relapse.

Four different CANTAB tests that assess different cognitive domains—the speed of response, spatial planning, episodic, and working memories were performed (13). Reaction time (RTI) test measures the subject's speed of response and movement to a visual target (13). Our results showed that the speed of response was better in healthy controls than MS patients, both relapse and stable groups. The significant improvement of response was found at 1 month after the first assessment in relapse group (Table 6). The impaired speed of response in MS patients was also reported in other studies based on different batteries and tests (24–26), yet still there are no any reports regarding differences in relapse and stable MS patients.

One touch stockings of Cambridge (OTS) test assesses the executive function (13). Relapse group performed the medium difficulty tasks of OTS test worse than the stable group and healthy persons group, while the performance in easy tasks didn't differ in all three study groups. The healthy persons performed the difficult task better than any MS patients' groups. It seems that easy tasks of OTS test is too simple both for healthy persons and MS patients and they are unable to distinguish the differences between these groups. Medium difficulty tasks may be informative to assess the changes between relapse and stable groups, though difficult tasks can help to distinguish differences only between MS patients and healthy persons (Table 3). Some studies have shown that the impairment of visuospatial functions is very common in MS patients and these functions were widely investigated in MS patients (27–29). Worse performance of visuospatial functions based on several assessments was established in MS patients by other authors (27–29) and even in patients with early MS (30) and clinically isolated syndrome (31). However, we could not find any data where the impairment of spatial planning was investigated during MS relapse. Assessing the changes of spatial planning after relapse in our study, the most important improvement of cognitive impairment was established in medium difficulty and easy tasks at 1 month, however, the results of the difficult task have shown that further improvement of spatial planning was also observed at 3 months after the first assessment (Table 7). It seems that the recovery of spatial planning after MS relapse is not over in 1 month and continues for at least 3 months after the relapse.

The Paired Associates Learning (PAL) test assesses episodic recall memory and new learning. This test is primarily sensitive to changes in medial temporal lobe functioning (13). We have found that medium difficulty tasks of episodic recall memory and learning, similarly to spatial planning assessment tasks, were performed worse by relapse group than by stable group and healthy controls. Difficult tasks were too hard to distinguish the differences of impairment in episodic visual memory between relapse and stable groups, they can only help to assess the differences between MS patients and healthy controls, while easy tasks could not help to distinguish any differences between all three groups (Table 4). Our findings have shown the impaired episodic memory and learning in MS patients, and are consistent with previous studies highlighting that one of the most commonly affected domains in MS patients is episodic memory and learning (32, 33). Computerized batteries have also demonstrated impaired episodic memory in MS patients (11, 33), however, still there lacks data regarding the impairment of episodic memory during MS relapse. In the present study, the improvement of episodic visual memory and learning after relapse was found at 1 month after the first assessment, and unlike the spatial planning tasks, no additional improvement was established at 3 months after the first assessment. It seems that episodic memory can significantly recover after MS relapse only during the first month after relapse treatment (Table 8). When comparing results of cognitive recovery after MS relapse based on OTS (= spatial planning) and PAL (episodic memory and learning) tests, a complex and uneven recovery pattern of different cognitive functions is evident.

The Spatial Working Memory (SWM) test is mainly used for assessment of working memory (13). When evaluating working memory in MS patients and controls, the same tendency of cognitive impairment as with spatial planning and episodic visual memory was established. Easy tasks of SWM test were too simple for MS patients, both with relapse and stable groups, and controls. Difficult tasks were quite hard for both MS patients' groups and did not benefit in assessing the changes between relapse and stable groups. Only tasks of medium difficulty were useful in distinguishing the impairment of working memory between relapse and stable groups (Table 5). Working memory is another widely investigated and commonly affected cognitive domain in MS patients (1, 2). The impairment of working memory in MS was proved based on different paper-pencil and computerized tests and batteries by other authors, yet still the impairment during relapse remains poorly understood and under-investigated (11, 34). Analysis of working memory after relapse has revealed a significant improvement of working memory at 1 month after the first assessment and an additional improvement was observed at 3 months after the first assessment (Table 9).

Our results confirm the results of other studies highlighting that different and multiple cognitive domains are impaired in MS patients. However, the impairment in MS is relatively less severe than is usually observed in primary neurodegenerative dementias (24, 25, 28, 29, 32, 33). In addition, in our study, the impairment of various cognitive domains was also observed in the stable group compared to controls, and in the relapse group, the impairment of various domains was even more significant during relapse.

An interesting finding is regarding cognitive changes after relapse. Usually, the end of the relapse and stabilization of physical relapse symptoms are considered to be at 1 month after relapse (20, 21). In our study, the improvement of most cognitive functions was also established during the first month after the first assessment, however, it seems that some cognitive functions were still improving further at least up to the third month. The results indicate that, perhaps, for accurate and exact assessment of cognitive stabilization after relapse, the assessment at 3 months after relapse would be appropriate.

### Clinical Implication

Relapsing Remitting MS is a disease characterized by very broad and considerable relapse heterogeneity in clinical presentation, cognitive dysfunction and responsiveness to treatments (1, 2, 20, 21). Cognitive assessment during remission and relapse should be an essential part of routine care for evaluation of the impact of new cognitive disorders. From the clinical and scientific point of view, it is useful to assess the neurocognitive status along with other aspects of neurological disability during MS relapse, and to improve the understanding of the clinically meaningful changes in cognition outcomes that may occur as a result of neurological worsening or response to treatment. Detection of cognitive impairment during relapse in MS patients is important as it allows appropriate support to be provided, possibilities of treatment and cognitive rehabilitation to overcome functional deficits.

### Limitations

Four CANTAB tests were selected for our study, however, some data suggests that other cognitive processes, such as verbal fluency, spatial relations, and others could also be affected in MS patients (35–37). It may be possible that the practice effect had the impact on the improvement of cognitive scores that was noted in relapsing MS patients. It would be beneficial to investigate other cognitive domains which could be impaired during MS relapse. In our study, all MS patients were on disease modifying therapy (DMT). There was no significant difference in the distribution of DMTs between MSr and MSr groups. The use of symptomatic medications which can cause some cognitive effects was limited by exclusion criteria. Data is lacking regarding test-retest reliability over 1 month of RTI, OTS, PAL, and SWM tests. It makes it problematic to estimate how much the improvement of test scores depends on the recovery after relapse, and what part of the betterment of scores may be related to the practice effect.

## Conclusions

The results of this study indicate that cognitive function is affected during MS relapse. The difficult task of CANTAB battery, which assesses the spatial planning, showed MS relapse related cognitive dysfunction. The changes in scores of episodic visual recall and working memory may be related to MS relapse. A significant improvement in the speed of response, spatial planning, episodic visual recall and working memory was established at 1 month after MS relapse. The additional improvement in spatial planning, for the most difficult task and working memory, was observed at 3 months after MS relapse. It may be possible that the practice effect had the impact on the improvement in cognitive scores that was noted in relapsing MS patients.

## Data Availability

All datasets generated for this study are included in the manuscript and/or the Supplementary Files.

## Ethics Statement

The study protocol was approved by Vilnius Regional Biomedical Research Ethics Committee (Approval No. 158200-13-644-191). All methods were performed in accordance with the relevant guidelines and regulations. Participants voluntary agreed to participate in the study and written informed consent (ICF) was obtained from all participants before inclusion in the study. Screening evaluation was started after the ICF was signed.

## Author Contributions

NG contributed to the concept and design of the study, acquisition of the data, analysis and interpretation of the data, and drafting of the manuscript. GK contributed to the concept and design of the study, analysis and interpretation of the data, and drafting of the manuscript.

### Conflict of Interest Statement

The authors declare that the research was conducted in the absence of any commercial or financial relationships that could be construed as a potential conflict of interest.
